# Growth and metabolism of *Beauveria bassiana* spores and mycelia

**DOI:** 10.1186/s12866-015-0592-4

**Published:** 2015-11-19

**Authors:** Hongxia Liu, Xusheng Zhao, Mingxin Guo, Hui Liu, Zhiming Zheng

**Affiliations:** Jujube Scientific Research and Applied Center, Life Science College, Luoyang Normal University, 471000 Luoyang, P. R. China; Key Laboratory of Ion Beam Bioengineering, Hefei Institutes of Physical Science, Chinese Academy of Sciences and Anhui Province, Hefei, Anhui 230031 P. R. China

**Keywords:** *Beauveria bassiana*, Gompertz model, PCA, HPLC-MC, Metabolism

## Abstract

**Background:**

Fungi are ubiquitous in nature and have evolved over time to colonize a wide range of ecosystems including pest control. To date, most research has focused on the hypocrealean genera *Beauveria bassiana*, which is a typical filamentous fungus with a high potential for insect control. The morphology and components of fungi are important during the spores germination and outgrow to mycelia. However, to the best of our knowledge, there is no report on the morphology and components of *B. bassiana* spores and mycelia. In the work, the growth and metabolism of *Beauveria bassiana* spores and mycelia were studied. High performance liquid chromatography-mass spectrometry (HPLC-MS) was employed to study the metabolism of *B. bassiana* spores and mycelia. Principal component analysis (PCA) based on HPLC-MS was conducted to study the different components of the spores and mycelia of the fungus. Metabolic network was established based on HPLC-MS and KEGG database.

**Results:**

Through Gompertz model based on macroscopic and microscopic techniques, spore elongation length was found to increase exponentially until approximately 23.1 h after cultivation, and then growth became linear. In the metabolic network, the decrease of glyoxylate, pyruvate, fumarate, alanine, succinate, oxaloacetate, dihydrothymine, ribulose, acetylcarnitine, fructose-1, 6-bisphosphate, mycosporin glutamicol, and the increase of betaine, carnitine, ergothioneine, sphingosine, dimethyl guanosine, glycerophospholipids, and in spores indicated that the change of the metabolin can keep spores in inactive conditions, protect spores against harmful effects and survive longer.

**Conclusions:**

Analysis of the metabolic pathway in which these components participate can reveal the metabolic difference between spores and mycelia, which provide the tools for understand and control the process of of spores germination and outgrow to mycelia.

## Background

Fungi are ubiquitous in nature and have evolved over time to colonize a wide range of ecosystems including pest control. Over the past century, approximately 1000 fungal species have been reported to kill insects [1–3]. To date, most research has focused on the hypocrealean genera *Beauveria bassiana*, which is a typical filamentous fungus with a high potential for insect control, because its spores are relatively easy and inexpensive to mass produce for field applications [4–6]. Moreover, it is known to have nontoxic effects on nontarget organisms, including animals and humans [7]. killing insect by fungi is a process of spore germination and outgrow to mycelia [8], which can be concluded as follows: as soon as a fungal spore is exposed to its favourable conditions, it changes from a dormant state to an actively metabolizing cell, and then a germ tube emerges from the spore. When the germ tube reaches a certain length, the spore is considered to be germinated. In the next steps, germ tube elongation and branching take place until the mycelium forms a colony [9]. The process has been assessed through typical reverse genetic gene-specific studies in terms of cell division [10], trehalose metabolism [11], respiration [12, 13], and nucleic and protein synthesis during spore germination [14]. We can conclude that the morphology and components of fungi during spores outgrowth to mycelia are changed [15, 16]. However, to the best of our knowledge, there is no report on the morphology and components of *B. bassiana* spores and mycelia. The change of morphology and components can reveal the connection between spores and mycelia, and provide a systems-level understanding of the cell.

Despite its importance, only a limited number of methodologies have been developed for morphology and components analysis. This is primarily due to the characteristics of most components that display high polarity, nonvolatility, poor detectability, and overall similar properties [17]. Recently, high performance liquid chromatography − mass spectrometry (HPLC-MS) equipped with electrospray ionization (ESI) detection has been used for components analysis [18–21]. It is a robust, sensitive, and selective technique, and also has become popular for quantitative and qualitative analyses. In the present study, the morphology of *B. bassiana* spores and mycelia were studied by combining macroscopic and microscopic techniques. And then HPLC-MS coupled with PCA were used to distinguish different metabolites of mycelia and spores. In addition, metabolic pathway was established based on HPLC-MS and KEGG database. Tracking metabolite changes under different conditions not only provides direct information on metabolism but is also complementary to gene expression and proteome analysis [22, 23]. Metabolomics, which can be defined as the measurement of the level of all intracellular metabolites, has become a powerful new tool for gaining insight into cellular function. The aim of the study was to reveal the reason of keep survive longer and infective of spores by compare significant change in metabolites between spores and mycelia. And provide the tools for understand and control the process of spores germination and outgrow to mycelia.

## Results and discussion

### Spore germination kinetics

The germination of spores takes place when the spores are introduced into a proper environment, which requires proper nutrition and special conditions. The spore germination can be divided into three phases: spore swelling, germ tube emergence and germ tube elongation [9]. In the first phase, spores begin to swell to increase their dormant diameter significantly until a germ tube emerges (second phase). The two phases in early growth are supported by mobilization and utilization of storage compounds in the spores. In the third phase the elongation of the germ tube is observed, which contributes to biosynthesis and extension by uptake and metabolism of nutrients from the medium [15].

**Fig. 1:**
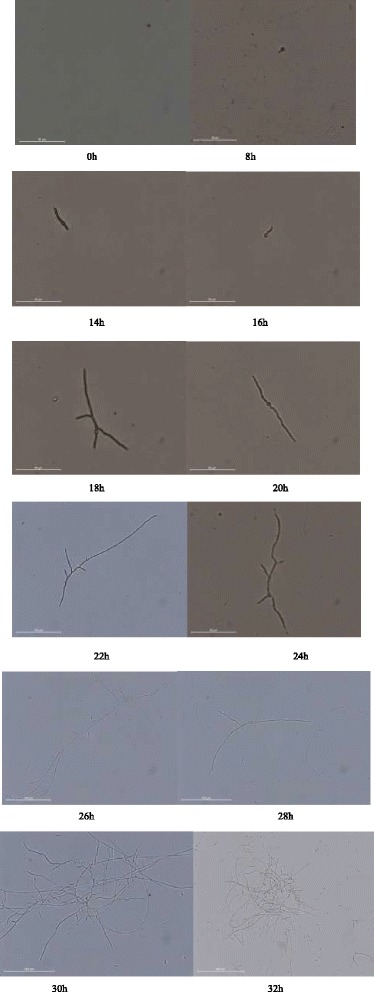
Spores germination and hyphal extendtion of *B. bassiana* in time on PDA at 26 °C via microscope (0–22 h: magnification × 640, 24–30 h: magnification × 320, 32 h magnification × 80)

The spore germination kinetics was investigated in the study. The values for hyphal length were measured with the aid of Image-Pro Plus software in a series of images monitoring the growth of *B. bassiana* spores on PDA at 26 °C, and the duration of the germination phase was estimated. Until the 6th hour of the cultivation, no germ tubes could be spotted, although an increase in the mean diameter of spores due to swelling. (Fig. 1).

Figure 1 showed typical forms of *B. bassiana* spores and hyphae in their development. Tubes emerged from 8 h to approximately 11 h. About 10 h after cultivation, most of the spores had their tubes emerged. At that moment the spores entered the phase of tube elongation. The hyphae remained unbranched till the 20th hour. After 28 h, the objects became difficult to observe and analyse due to their increasing concentration and aggregation of hyphal elements.

**Fig. 2:**
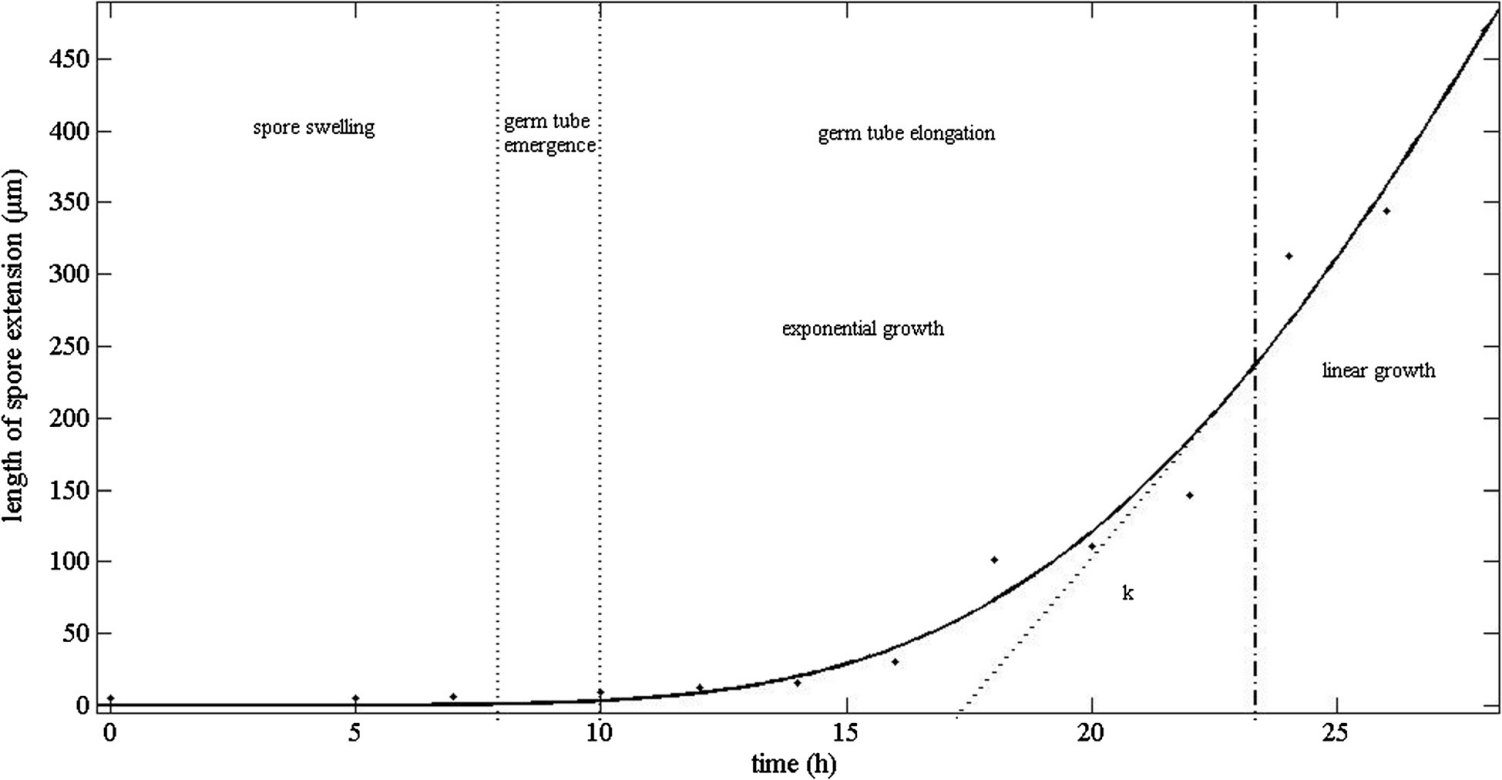
Germination kinetics of *B. bassiana* spores on PDA at 26 °C, the lines indicate the fitting of the Gompertz model

Models were used to describe the behavior of microorganisms [24]. In the study, the modified Gompertz model was employed to detect the relation between spore elongation length and culture time, which can provide useful information about the variance of the germination time for individual spores. Figure 2 showed changes of the most important morphological parameters. It can be seen that the modified Gompertz equation described satisfactorily the length of spore elongation over time for *B. bassiana*, with coefficients of determination (R^2^) of 0.9825. The three phases, spore swelling, germ tube emergence and germ tube elongation can be observed distinctly. In the phase of germ tube emergence and in the early hyphal development, the measured length appeared to increase exponentially until approximately 23.1 h after cultivation, when growth became linear. The above results are in agreement with the findings of Trinci [25, 26], who examined the kinetics of hyphal extension of several fungi, and considered the observed transition from exponential to linear growth can be attributed to the weakness of the hyphal tips to incorporate the increasing material that is being supplied or the deficiency of transporting material from distal hyphal regions.

### Principal component analysis

PCA based on HPLC-MS data was used to study the metabolomic differences of mycelia and spores extracts from *B. bassiana*. PCA is an unsupervised pattern recognition method, which means that no prior knowledge concerning groups or tendencies within the data sets is necessary. PCA is usually employed to reduce the dimensionality of the data and extract essential information from large, mixed data sets [27]. A score plot is applied for the grouping of samples by reducing the dimensionality of the data. The complex data was reduced to two principal components PC1 and PC2, which can represent most of the components. The values of the two variables for the observations are called factor scores*,* two factors scores can be interpreted geometrically as the projections of the observations onto the whole components. The data sets exhibiting similarities are clustered together, and those that are different are placed further apart [28].

**Fig. 3:**
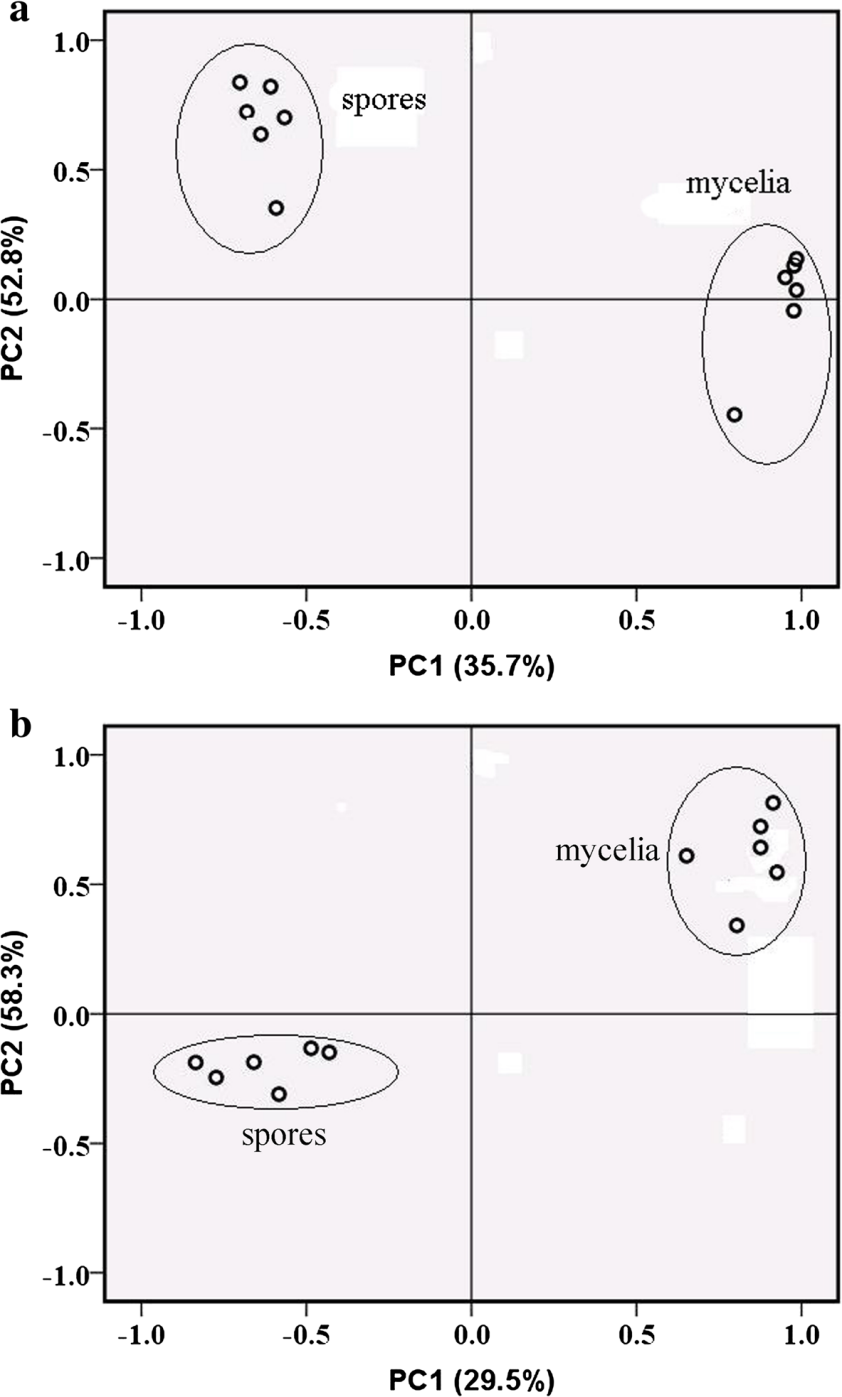
PCA score plots derived from HPLC − MS spectra of mycelia and spores extracts in positive mode (**a**) and negative mode (**b**)

**Fig. 4:**
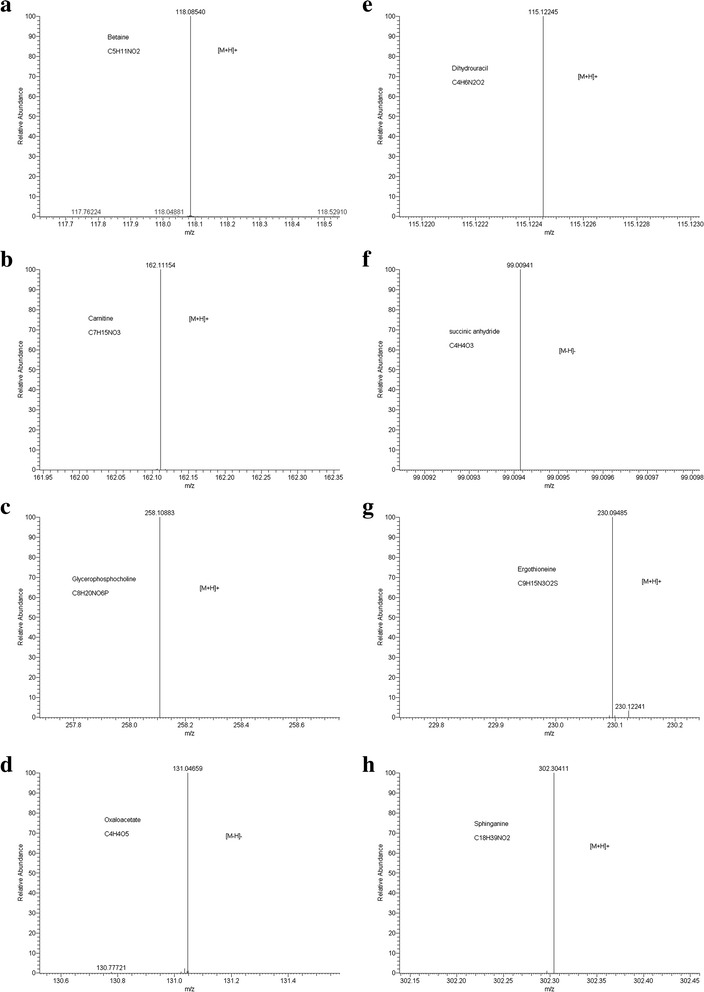
MS spectrum of the eight compounds (**a**−**d** in mycelia extracts, **e**−**h** in spores extracts). **a** betaine, **b** carnitine, **c** glycerophosphocholine, **d** oxaloacetate, **e** dihydrouracil, **f** succinic anhydride, **g** ergothioneine, **h** sphinganine

Acid compounds were extracted in negative mode, while alkaline compounds were extracted in positive mode, to study the whole components extracted from spores and mycelia, both positive mode and negative mode must be employed to PCA. As shown in Fig. 3, from both positive mode and negative mode, PCA score plots employed in this study found that the mycelia were clearly separated from the spores of *B. bassiana*, and the spores or mycelia were grouped correctly together respectively. In the posotive mode, both principle components were significant: PC1 accounts for 35.7 % of the total variance and PC2 accounts for 52.8 %. The first two principal components (PC1 and PC2) explain more than 80 % of the total variance. In the negative mode, PC2 leads to classification of the two groups and accounts for 58.3 % of the total variance, whereas PC1 denotes 29.5 % of the total variance. The high-resolution ESI-MS data for some metabolites are shown in Fig. 4. These findings were in good agreement with HPLC–MS based metabolic profiles (Tables 1 and 2). In Table 1, twenty-eight major components were identified from *B. bassiana* mycelia by HPLC-MS method, eleven of which were different from that in spores, such as glycerophosphocholine, palmitic acid, linoleic acid, phosphatidylethanolamine. For spores, thirty-six compounds were extracted, seventeen of which were distinguished from mycelia, such as succinic anhydride, dihydrouracil, mannitol, sphinganine (Table 2).

**Table 1:** Metabolites putatively identified by HPLC − MS in mycelia extracts

RT (min)	Detected mass	Metabolite	Ionization mode	Molecular formula	Theoretical mass	Δmass (mDa)	Relative concent-ration
3.23	72.99348	Glyoxylate	ESI (−)	C_2_H_2_O_3_	74.00039	1.460	↑
64.92	87.00903	Pyruvate	ESI (−)	C_3_H_4_O_3_	88.01739	−15.625	↑
1.32	90.05431	Alanine	ESI (+) and ESI (−)	C_3_H_7_NO_2_	89.0932	−0.645	↑
5.70	115.05154	Fumarate	ESI (−)	C_4_H_4_O_4_	116.07216	0.758	↑
2.82	117.01965	Succinate	ESI (−)	C_4_H_6_O_4_	118.08804	1.415	↑
1.45	118.08540	Betaine	ESI (+)	C_5_H_11_NO_2_	117.07704	−0.855	↓
1.17	129.13795	Dihydrothymine	ESI (+)	C_5_H_8_N_2_O_2_	128.12922	0.952	+
1.30	131.04636	Oxaloacetate	ESI (−)	C_4_H_4_O_5_	132.07156	−1.023	↑
45.75	149.04712	Ribulose	ESI (−)	C_5_H_10_O_5_	150.55480	1.899	↑
1.30	162.11154	Carnitine	ESI (+)	C_7_H_15_NO_3_	161.10318	−0.930	↓
1.45	204.12184	Acetylcarnitine	ESI (+)	C_9_H_17_NO_4_	203.11348	−1.195	↑
24.58	230.11407	Ergothioneine	ESI (+)	C_9_H_15_N_3_O_2_S	229.30031	0.569	↓
41.61	255.23244	Palmitic acid	ESI (−)	C_16_H_32_O_2_	256.24080	1.677	+
1.35	258.10883	Glycerophosphocholine	ESI (+)	C_8_H_20_NO_6_P	257.10047	−1.270	+
39.18	279.23239	Linoleic acid	ESI (−)	C_18_H_32_O_2_	280.24075	0.533	+
29.99	295.22723	8-hydroxy-linoleic acid	ESI (−)	C_18_H_32_O_3_	296.23559	−0.458	+
1.32	296.06451	5-aminoimidazole ribonucleotide	ESI (+)	C_8_H_14_N_3_O_7_P	295.05615	0.297	+
59.15	309.27618	Linoleic acid ethyl ester	ESI (+)	C_20_H_36_O_2_	308.26782	−0.080	+
28.48	312.30252	Dimethyl guanosine	ESI (+)	C_12_H_17_N_5_O_5_	311.29388	1.726	↓
1.24	317.11334	Mycosporin glutamicol	ESI (+)	C_13_H_20_N_2_O_7_	316.10498	1.278	↑
3.37	335.07434	Fructose −1,6-bisphosphate	ESI (−)	C_6_H_10_O_12_P_2_	336.08392	1.500	↑
28.53	476.27719	Phosphatidylethanolamine (18:2/0:0)	ESI (−)	C_23_H_44_NO_7_P	477.28555	−0.024	+
30.57	478.29260	Phosphatidylethanolamine (18:1/0:0)	ESI (−)	C_23_H_46_NO_7_P	479.30096	0.095	+
35.26	522.35986	Phosphotidylcholine (18:1/0:0)	ESI (+)	C_26_H_52_NO_7_P	521.3515	0.154	↓
36.15	524.29797	Phosphatidyserine (18:1/0:0)	ESI (+)	C_24_H_46_NO_9_P	523.28961	−0.082	↓
36.80	595.28802	Phosphatidylinositol (18:2/0:0)	ESI (−)	C_27_H_49_O_12_P	596.29638	0.230	↓
40.74	597.30420	Phosphatidylinositol (18:1/0:0)	ESI (−)	C_27_H_51_O_12_P	598.31256	0.760	↓
16.52	631.38678	Beauverolide Ka	ESI (+)	C_37_H_50_N_4_O_5_	630.378411	−0.345	+

↑ Relative concentration of the component was higher in mycelia than in spores; ↓, Relative concentration was lower in mycelia than in spores; +, Relative concentration was only found in mycelia, and not found in spores

**Table 2:** Metabolites putatively identified by HPLC − MS in spores extracts

RT (min)	Detected mass	Metabolite	Ionization mode	Molecular formula	Theoretical mass	Δmass (mDa)	Relative concent-rations
2.95	72.99202	Glyoxylate	ESI (−)	C_2_H_2_O_3_	74.00039	−1.530	↓
64.87	87.00903	Pyruvate	ESI (−)	C_3_H_4_O_3_	88.01739	−1.360	↓
1.30	90.05455	Alanine	ESI (+) and ESI (−)	C_3_H_7_NO_2_	89.0932	−0.405	↓
2.95	99.00914	Succinic anhydride	ESI (−)	C_4_H_4_O_3_	100.01751	1.470	+
5.79	115.07640	Fumarate	ESI (−)	C_4_H_4_O_4_	116.07216	−1.627	↓
1.17	115.12245	Dihydrouracil	ESI (+)	C_4_H_6_N_2_O_2_	114.10264	0.361	+
2.98	117.07478	Succinate	ESI (−)	C_4_H_6_O_4_	118.08804	−0.002	↓
1.34	118.08559	Betaine	ESI (+)	C_5_H_11_NO_2_	117.07704	−0.024	↑
45.76	131.06079	Oxaloacetate	ESI (−)	C_4_H_4_O_5_	132.07156	1.486	↓
3.32	128.03557	Pyroglutamic acid	ESI (−)	C_5_H_7_NO_3_	129.04393	1.350	+
1.05	129.13797	Dihydrothymine	ESI (+)	C_5_H_8_N_2_O_2_	128.12922	−0.085	↓
2.27	146.04608	Glutamic acid	ESI (−)	C_5_H_9_NO_4_	147.05444	1.296	
64.65	149.04669	Ribulose	ESI (−)	C_5_H_10_O_5_	150.05505	1.240	↓
1.28	162.11182	Carnitine	ESI (+)	C_7_H_15_NO_3_	161.10318	−0.930	↑
1.23	173.10434	Arginine	ESI (−)	C_6_H_14_N_4_O_2_	174.1127	1.038	+
1.34	181.07187	Mannitol	ESI (−)	C_6_H_14_O_6_	182.08023	1.205	+
1.43	204.12219	Acetylcarnitine	ESI (+)	C_9_H_17_NO_4_	203.11348	−0.845	↓
1.38	230.09485	Ergothioneine	ESI (+)	C_9_H_15_N_3_O_2_S	229.30031	1.203	↑
1.37	236.14839	Ulvaline	ESI (+)	C_10_H_21_NO_5_	235.14003	−0.859	+
33.64	299.25864	3-hydroxy-stearic acid	ESI (−)	C_18_H_36_O_3_	300.267	0.569	+
27.63	302.30411	Sphinganine	ESI (+)	C_18_H_39_NO_2_	301.29575	−1.246	↑
2.38	312.12906	Dimethyl guanosine	ESI (+)	C_12_H_17_N_5_O_5_	311.1207	−0.025	↑
3.32	315.11945	Mycosporin glutamicol	ESI (−)	C_13_H_20_N_2_O_7_	316.12781	0.776	↓
24.46	318.29892	2-amino-6-methyl-1,3,4-heptadecanetriol	ESI (+)	C_18_H_39_NO_3_	317.29056	−1.351	+
30.58	330.33508	2-amino-1,3-eicosanediol	ESI (+)	C_20_H_43_NO_2_	329.32672	−1.576	+
2.81	335.07434	Fructose −1,6-bisphosphate	ESI (−)	C_6_H_10_O_12_P_2_	336.08392	3.0	↓
27.79	346.32990	2-amino-1,3,4-eicosanetriol	ESI (+)	C_20_H_43_NO_3_	345.32154	−1.671	+
41.75	433.23291	Phosphatidic acid (18:2/0:0)	ESI (−)	C_21_H_39_O_7_P	434.24127	2.056	+
43.20	435.25119	Phosphatidic acid (18:1/0:0)	ESI (−)	C_21_H_41_O_7_P	436.25955	−2.939	+
31.79	520.33716	Phosphotidylcholine (18:2/0:0)	ESI (+)	C_26_H_50_NO_7_P	519.32880	1.589	+
34.49	522.35303	Phosphotidylcholine (18:1/0:0)	ESI (+)	C_26_H_52_NO_7_P	521.34467	−2.386	↑
25.76	524.29108	Phosphatidyserine (18:1/0:0)	ESI (+)	C_24_H_46_NO_9_P	523.28272	1.724	↑
45.33	538.31598	Phosphatidyserine (19:0/0:0)	ESI (−)	C_25_H_50_NO_9_P	539.32434	2.501	+
16.13	566.34436	Phosphatidyserine (21:0/0:0)	ESI (−)	C_27_H_54_NO_9_P	567.35272	2.515	+
11.48	595.28656	Phosphatidylinositol (18:2/0:0)	ESI (−)	C_27_H_49_O_12_P	596.29492	−1.230	↑
67.34	597.30200	Phosphatidylinositol (18:1/0:0)	ESI (−)	C_27_H_51_O_12_P	598.31036	−1.440	↑

↑, Relative concentration of the component was higher in spores than in mycelia; ↓, Relative concentration was lower in spores than in mycelia; +, Relative concentration was only found in spores, and not found in mycelia.

### Metabolic pathway analysis

Nutritional limitations or increase in cell density leads *B. bassiana* to produce dormant, environmentally resistant spores. The complex morphological changes that occur during sporulation are thought to be highly controlled by metabolic networks [29]. However, no comprehensive metabolite profiling approach has been used to demonstrate alterations in large-scale metabolites. Thus, to overall unravel the effects of these metabolites on the metabolic network, HPLC-MS described above was employed to detect the metabolic pathway of some metabolites identified in mycelia and spores.

**Fig. 5:**
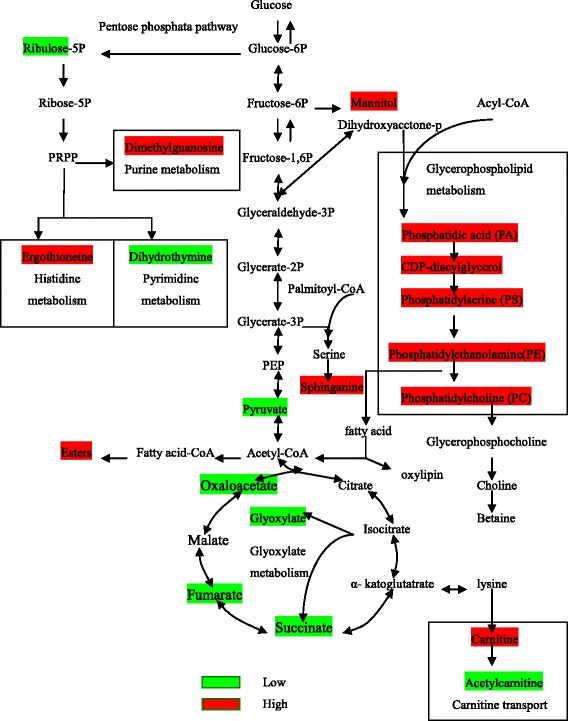
General biosynthetic pathways of some metabolites in *B. bassiana* according to the HPLC-MS and KEGG database. Green boxes indicate metabolites whose levels lower in spores than in mycelia. White red boxes represent the levels of metabolites higher in spores than in mycelia. Abbreviations: PRPP, phosphoribosyl pyrophosphate; PEP, phosphopyruvate

*B. bassiana* cells produce spores under conditions of nutrition deprivation. Interestingly, most metabolites (pyruvate, fumarate and ribulose) in the glycolytic, tricarboxylic acid (TCA) cycle and pentose phosphate pathways were markedly decreased in the stage of spores (Fig. 5). In particular, the level of fructose-1, 6-bisphosphate, a key factor in catabolite repression, dropped more rapidly in spores than in mycelia. It was possibly because that the decrease in fructose-1, 6-bisphosphate resulted in slow of metabolism, which kept spores in a dormant, metabolically inactive conditions and help them survive longer.

The pentose phosphate pathway is a biochemical pathway parallel to glycolysis, which can generates NADPH and pentoses (5-carbon sugars). While it does involve in oxidation of glucose, its primary role is anabolic rather than catabolic [30, 31]. The pathway is a major source of reductant for biosynthetic processes such as fatty-acid synthesis and assimilation of inorganic nitrogen [32], and maintains redox potential necessary to protect against oxidative stress [33]. In the first stage of pentose phosphate pathway, glucose-6-phosphate was converted into ribose 5-phosphate, along with the energy production. And then ribose 5-phosphate was converted to phosphoribosyl pyrophosphate (PRPP). At the step, the metabolism was divided into three different pathways: histidine metabolism, purine metabolism and pyrimidine metabolism (Fig. 5). In the histidine metabolism, ergothioneine was extracted from *B. bassiana* as a metabolin, which is a naturally occurring amino acid and a thiourea derivative of histidine only produced by fungi and some prokaryotes. Ergothioneine has antioxidant properties in vitro [34]. Under laboratory conditions, it scavenges hydroxyl radicals and hypochlorous acid, inhibits production of oxidants by metal ions, and may participate in metal ion transport and regulation of metalloenzymes [35, 36]. In 2012, Bello identified an ergothioneine biosynthetic gene *Egt-1* in *Neurospora crass* and demonstrated that ergothioneine enhanced conidial survival and protected against peroxide toxicity during spore germination [37]. Thus, the high levels of ergothioneine in the spores play an antioxidant role, and keep active of spores.

Dimethyl guanosine is a product of purine metabolism. Charles and his co-workers have mentioned that dimethyl guanosine as a self-inhibitor serves to regulate spore development in *Dictyostelium mucoroides* [38]. Thus, the increased dimethyl guanosine in spores might diminish some gene transcription, which would make metabolism slower [39], and lead to improved shelf life of biological insecticide.

Dihydrothymine is a degradation product of thymine in the pyrimidine metabolism which is catalyzed by dihydropyrimidine dehydrogenase [40]. It associated with valine, leucine and isoleucine metabolism [41]. The decrease of dihydrothymine in spores might mean that the correlative metabolism network became slower than that in mycelia.

A mannitol cycle was first proposed by Hult and Gatenbeck [42], which comprises four enzymes: mannitol 1-phosphate dehydrogenase (MPDH), mannitol 1-phosphate phosphatase (MPP), NADP^+^-mannitol 2-dehydrogenase (MDH), and hexokinase (HX), of which MPDH is the main synthetic enzyme and MDH is the main catabolic enzyme. The roles of mannitol vary in different fungi and might act as a scavenger of reactive oxygen species [43]. It was found only in spores (not found in mycelia) of *B. bassiana*, which implied that mannitol in spores might have an important impact on environment adaptability, germination, and virulence.

Glycerophospholipids, as glycerol-based phospholipids, are the main components of biological membranes, which play an important role in the generation of both extracellular and intracellular signals. As showed in Fig. 5, the increase of glycerophospholipids, esters and carnitine and the decrease of glyoxylate, pyruvate and acetylcarnitine in spores indicated that the hydrolysis of lipids and oxidation of fatty acids were depressed [44].

Beauverolides are insecticidal cyclodepsipeptides in *B. bassiana* [45]. The emergence of beauverolide Ka in mycelia (Table 1) may be related to the oxidative stress and/or oxylipin metabolism.

Sphingosine is a signaling lipid and may be involved in insect-fungi recognition, which can recognize the receptors on spores and induce the germination [46]. Sphinganine begins with the condensation of serine with palmitoyl-CoA to form 3-ketosphinganine, which is rapidly reduced to sphinganine [47]. Sphinganine is the biosynthetic precursor of sphingosines and sphingolipids, and its emergence in spores may affect fungal signaling and germination.

The TCA is a key metabolic pathway that unifies carbohydrate, fat and protein metabolism. The reactions of the cycle are carried out by eight enzymes that completely oxidize acetyl-CoA into two molecules of carbon dioxide. Through catabolism of sugars, fats and proteins, a two-carbon organic product acetate in the form of acetyl-CoA is produced which enters the citric acid cycle [48]. An increase of carnitine and decrease of acetylcarnitine, succinate, oxaloacetate and fumarate suggested that the TCA was depressed and lipid metabolism was enhanced in the spores. As a result, lipid was accumulated to protect spores against harmful effects of environment or others.

## Conclusion

The proposed HPLC-MS methods enable the global determination of charged species, so that they can be used as universal tools for metabolome analysis. Metabolome data, along with macroscopic and microscopic techniques were used to provide important new information of the metabolism and growth of *B. bassiana* spores and mycelia. Gompertz model based on macroscopic and microscopic techniques was used to detect the relation between spores elongation length and culture time. Spore elongation length was found to increase exponentially until approximately 23 h after cultivation, and then growth became linear. The results of PCA displayed clear differences of the components in mycelia and spores of the fungus. Metabolic pathway of *B. bassiana* spores and mycelia was established based on HPLC-MS and KEGG database, which revealed the presence of twenty-eight major components in mycelia and thirty-six compounds in spores. In the metabolic network, the decrease of glyoxylate, pyruvate, fumarate, alanine, succinate, oxaloacetate, dihydrothymine, ribulose, acetylcarnitine, fructose-1, 6-bisphosphate, mycosporin glutamicol, and the increase of betaine, carnitine, ergothioneine, sphingosine, dimethyl guanosine, glycerophospholipids, and in spores indicated that the change of the metabolin can keep spores in inactive conditions, protect spores against harmful effects and survive longer. The study provided the tools for understand and control the process of spores germination and outgrow to mycelia.

## Methods

### Microorganisms and culture medium

*B. bassiana* Bb0062 provided by the Anhui Provincial Key Laboratory of Microbial Control, Anhui Agricultural University, was stored at −20 °C in sterilized cryovials containing 10 % glycerol (in 0.02 % Tween 80 solution). It was cultured on potato dextrose agar (PDA) slants (potato 200 g/L, glucose 20 g/L and agar 20 g/L) at 26 °C for 7 days, and then stored at 4 °C until use.

### Preparation of inocula

*B. bassiana* was cultured on PDA medium mentioned above at 26 °C for 7 days to obtain heavily sporulating cultures. Spores were then suspended in sterile distilled water containing 0.02 % (v/v) Tween 80 by gently scraping the agar surface with a sterile spatula, and then filtered through two layers of gauze to remove any debris (mostly mycelial fragments). The final spore concentration was adjusted to 1 × 10^6^ spores/mL and used as quickly as possible.

### Assessment of germination and outgrowth

Portions (100 μL) of the inoculum (*B. bassiana*), containing approximately 10^5^ spores, were surface plated aseptically on PDA. After inoculation, plates were sealed with parafilm to prevent moisture loss, and stored at 26 °C for 2 days. To monitor the kinetic behavior of the *B. bassiana* spores for prolonged periods (from germination to mycelium formation), computer morphometry (Leica Microscopy System Ltd, DMI 4000B, Germany) was employed to examine spore germination and mycelia outgrowth. Germination time was defined as the time at which the length of the germ tube was equal to the diameter of the swollen spore. Samples were measured every 2 h until 32 h. Images was analysed by Image-Pro Plus image analysis software version 6.3 (MediaCybernetics Inc., Bethesda, United States) and an auto-focus system.

For the germination study, length of germ tube over time was fitted to the modified Gompertz equation (Eq. (1)) [49] for the estimation of the germination kinetic parameters (ke and c):

1 $$ L={L}_{\max}\kern0.5em  \exp \left(- \exp \left[\frac{ke}{L_{\max }}\left(c\mathit{\hbox{-}}t\right)+1\right]\right) $$

where t (h) is the time, L indicates length of spores elongation at time t, L_max_ represents length of hypha t → +∞, ke signifies the slope of tangent line through the inflection point, c is the time when hyphal extention reach to maximum speed.

### Mycelia and spores preparation for HPLC-MS analysis

Mycelia were harvested after 32 h of growth by a scoop, suspended in sterile water, and then filtered through two layers of gauze to remove debris, subsequently frozen in liquid nitrogen to terminate metabolism, and kept at −80 °C. Spores were collected at 7 days post-inoculation by scrapping the colony into 0.01 % Tween 80 solution, and the contents were vortexed and filtered through a 10 μm microfiltration membrane to remove any debris. The filtrate was transferred to a 50 mL centrifuge tube and centrifuged for 10 min at 8000 rpm. The precipitate was resuspended in 2 mL of distilled water and transferred to a 5 mL centrifuge tube, centrifuged for 10 min at 10,000 rpm, and then frozen in liquid nitrogen to terminate metabolism. Finally, the spores were kept at −80 °C. Three independent biological replicates were measured per assay.

Mycelia and spores were lyophilized until a constant weight was attained. Mycelia were then crushed into a fine powder and then kept at 4 °C until extraction. Ten milligrams of sample was extracted with 2 mL of 80 % methyl alcohol (Tedia company, USA, HPLC grade), followed by 1 min of vortexing and subsequent sonication (12-KHz, 8-s exposure followed by a 4-s rest interval) for 1 h. Samples were further kept at 4 °C for 12 h in the dark. After centrifugation at 8000 rpm for 10 min, 1.8 mL of supernatant was collected and dried with a centrifugal concentrator. All samples were stored at −80 °C until analysis. The dried extracts were redissolved ultrasonically in 300 μL of 80 % methanol and filtered through a 0.22 μm polyvinylidene fluoride membrane filter before HPLC − MS analysis.

### HPLC − MS conditions

In order to determine retention time and to obtain extract HPLC profiles, HPLC was performed on a Thermo-Fisher UPLC system (Thermo-Fisher, SanJose, CA, USA) coupled with an LTQ XL mass spectrometer. HPLC analyses were conducted on a C18 reversed-phase (RP) column (5 μm, 3 mm × 150 mm, 100A, Luna PFP Phenomenex, Torrance, CA, USA). The parameters were as follows: injection volume, 5 μL; column temperature, 40 °C; flow rate, 0.3 mL/min; and the eluates were monitored by full-length scan from 200 to 600 nm. The mobile phase was (A) 0.1 % formic acid (Anaqua Chemical Supply, USA, HPLC grade) in water and (B) 0.1 % formic acid in acetonitrile (Merck, Germany, HPLC grade), and gradient elution was carried out: 5 % B for 0–3 min, 5–100 % B for 3–50 min, and 100 % B for 50–60 min. The mass spectrometer parameter settings used for the measurement were as follows: ionization mode, for both positive and negative; gas temperature, 350 °C; drying gas, 12 L/min; nebulizer pressure, 45 psi; capillary voltage, 4000 V in positive mode and 3500 V in negative mode; fragmentor voltage, 215 V in positive mode and 170 V in negative mode; skimmer voltage, 60 V; and OCT 1 RF, 250 V. Data acquisition was performed in the m/z range of 50–1100 Da.

### Data processing and statistical analysis

All data were processed using the Xcalibur software provided by the manufacturer. After all of the detected peaks were subjected to noise-reduction in both the HPLC and MS domains, the analytical peaks were processed by the software. A list of peak intensities with retention times and m/z data pairs was generated. The intensity of each peak was normalized by the sum of all of the peak intensities. Peaks with signal-to-noise (S/N) ratios lower than 5 were rejected. PCA were performed by SPSS v18.0 (IBM SPSS Statistics, Ontario, Canada) to envisage the different components of *B. bassiana* spores and mycelia.

### Metabolite identification

The small-molecule inventory (SMI) or metabolome is a pattern of molecules that reflects the cell’s status. The molecular formula calculated by the Xcalibur software was predicted, and based on a general understanding of fungi metabolism pathways by searching web databases (Dictionary of Natural Products, METLIN, PUBCHEM, and CHEMSPIDER). The exact monoisotopic masses of possible metabolites were calculated based on their elemental compositions. Putative biomarkers were verified by its elution order (polarity) and structure characteristics. The ambiguous metabolites were identified by comparison to authentic compounds available or referring to the published literature about fungi, especially entomopathogenic fungi.

## Abbreviations

ESI: Electrospray ionization
HPLC-MS: High performance liquid chromatography-mass spectrometry
HX: Hexokinase
KEGG: Kyoto Encyclopedia of Genes and Genomes
MDH: Mannitol 2-dehydrogenase
MPDH: Mannitol 1-phosphate dehydrogenase
MPP: Mannitol 1-phosphate phosphatase
PCA: Principal component analysis
PEP: phosphopyruvate
PRPP: Phosphoribosyl pyrophosphate
SMI: Small-molecule inventory
TCA: Tricarboxylic acid
